# Deep Learning-Based Deep Brain Stimulation Targeting and Clinical Applications

**DOI:** 10.3389/fnins.2019.01128

**Published:** 2019-10-24

**Authors:** Seong-Cheol Park, Joon Hyuk Cha, Seonhwa Lee, Wooyoung Jang, Chong Sik Lee, Jung Kyo Lee

**Affiliations:** ^1^Department of Neurosurgery, Seoul Metropolitan Government – Seoul National University Boramae Medical Center, Seoul, South Korea; ^2^Department of Neurosurgery, Gangneung Asan Hospital, University of Ulsan, Gangneung, South Korea; ^3^School of Medicine, Inha University, Incheon, South Korea; ^4^Department of Bio-Convergence Engineering, College of Health Science, Korea University, Seoul, South Korea; ^5^Department of Neurology, Gangneung Asan Hospital, University of Ulsan, Gangneung, South Korea; ^6^Department of Neurology, Asan Medical Center, University of Ulsan, Seoul, South Korea; ^7^Department of Neurosurgery, Asan Medical Center, University of Ulsan, Seoul, South Korea

**Keywords:** deep learning, deep brain stimulation, convolutional neural network, semantic segmentation, clinical application

## Abstract

**Background:**

The purpose of the present study was to evaluate deep learning-based image-guided surgical planning for deep brain stimulation (DBS). We developed deep learning semantic segmentation-based DBS targeting and prospectively applied the method clinically.

**Methods:**

T2^∗^ fast gradient-echo images from 102 patients were used for training and validation. Manually drawn ground truth information was prepared for the subthalamic and red nuclei with an axial cut ∼4 mm below the anterior–posterior commissure line. A fully convolutional neural network (FCN-VGG-16) was used to ensure margin identification by semantic segmentation. Image contrast augmentation was performed nine times. Up to 102 original images and 918 augmented images were used for training and validation. The accuracy of semantic segmentation was measured in terms of mean accuracy and mean intersection over the union. Targets were calculated based on their relative distance from these segmented anatomical structures considering the Bejjani target.

**Results:**

Mean accuracies and mean intersection over the union values were high: 0.904 and 0.813, respectively, for the 62 training images, and 0.911 and 0.821, respectively, for the 558 augmented training images when 360 augmented validation images were used. The Dice coefficient converted from the intersection over the union was 0.902 when 720 training and 198 validation images were used. Semantic segmentation was adaptive to high anatomical variations in size, shape, and asymmetry. For clinical application, two patients were assessed: one with essential tremor and another with bradykinesia and gait disturbance due to Parkinson’s disease. Both improved without complications after surgery, and microelectrode recordings showed subthalamic nuclei signals in the latter patient.

**Conclusion:**

The accuracy of deep learning-based semantic segmentation may surpass that of previous methods. DBS targeting and its clinical application were made possible using accurate deep learning-based semantic segmentation, which is adaptive to anatomical variations.

## Introduction

Deep learning is a machine learning technique using neural networks with multiple layers that are partly similar to a biological brain (Marcus, 2018). The majority of medical deep learning analyses are performed for diagnostic purposes rather than for surgical planning (Choi and Jin, 2016; De Fauw et al., 2018; Naylor, 2018). In a recent review and study, image-guided surgery was considered as a potential application of deep learning (Mehrtash et al., 2017; Naylor, 2018). However, the clinical application of deep learning in surgical planning remains almost unexplored. In the present study, we demonstrate deep learning-based surgical planning for deep brain stimulation (DBS) and its clinical application.

Current clinical approaches to surgical planning for DBS rely on imaging interpretation and processing, including magnetic resonance imaging (MRI)-based direct targeting (Holl et al., 2010; Foltynie et al., 2011; Aviles-Olmos et al., 2014) and image fusion techniques using co-registration with computed tomography (CT) and 1.5T and 3T MRI (Neumann et al., 2015). Atlas-based coordinates can be used when visualization of targets is insufficient, or to improve accuracy (Kochunov et al., 2002). Direct targeting using deep learning-based image analysis may be possible when target visualization is achieved with sufficient resolution. Later, electrophysiologic information, including microelectrode recording and intraoperative stimulation effects, can be considered for final decisions regarding electrode positions (Hariz, 2002; Amirnovin et al., 2006; Park et al., 2017). Here, we demonstrate deep learning-based surgical planning for DB for the first time to our knowledge. We investigated DBS in the subthalamic nucleus (STN) and in the posterior subthalamic area, a closely related target (Plaha et al., 2008; Blomstedt et al., 2009, 2010). The STN is an important target for Parkinson’s disease (Bejjani et al., 2000; Foltynie et al., 2011; Aviles-Olmos et al., 2014) and the posterior subthalamic area is an effective target for essential tremor (Plaha et al., 2008; Blomstedt et al., 2009, 2010).

Semantic segmentation results in pixel-wise image classification into predetermined classes, for example, anatomical structures (Mehta and Sivaswamy, 2017; Shelhamer et al., 2017). Semantic segmentation combines classification information (“what”) and location information (“where”) from image data (Shelhamer et al., 2017). Semantic segmentation can classify and identify the margins of multiple types of objects (Shelhamer et al., 2017). Recent convolutional neural network segmentation studies have not been optimized for the STN or red nucleus but instead for basal ganglia structures (Mehta and Sivaswamy, 2017; Milletari et al., 2017) or the striatum (Choi and Jin, 2016) only. Therefore, deep learning-based semantic segmentation of the STN and red nucleus is not well investigated. Thus, the current state-of-the-art methods for STN and red nucleus semantic segmentation are non-deep learning methods (Kim et al., 2019; Shamir et al., 2019).

Automatic targeting methods for DBS have potential clinical utility and may be non-inferior to manual methods (Pallavaram et al., 2015). However, the clinical application of automatic targeting methods is still under investigation (Pallavaram et al., 2015). In previous automatic methods, wide and robust applicability may have been limited due to the high variability of the STN anatomy (Naylor, 2018). We found that deep learning-based semantic segmentation shows highly accurate adaptability to considerable variations in STN shape, which enables real-world clinical application.

In the present study, we show the earliest cases of deep learning-based surgical planning. The results support the concept that accurate, deep learning-based semantic segmentation and surgical planning can be applied safely and successfully for DBS in clinical practice.

## Materials and Methods

### Institutional Approval

This study was pre-approved by the institutional review boards of Gangneung Asan Hospital (2018-07-22) and Asan Medical Center (S2016-1230-0005). Before clinical application, all patients and families signed informed consent forms that had been approved by the institutional review boards.

### Deep Learning and Imaging Methods

Training datasets were collected from patients who had undergone the DBS procedure and evaluation at Asan Medical Center between April 2014 and September 2017 (Park et al., 2017, 2018). Training and validation data were generated by using 3-Tesla (3T) T2^∗^ fast gradient-echo MRI sequences with a repetition time (TR) of 1026.3 ms, an echo time (TE) of 25 ms, and a flip angle of 30°. The field of view was anterior to posterior (AP) (mm) = 192, right to left (RL) (mm) = 192, and foot to head (FH) (mm) = 70. The voxel size was 0.375 mm, and the matrix size was 512.

**FIGURE 1:**
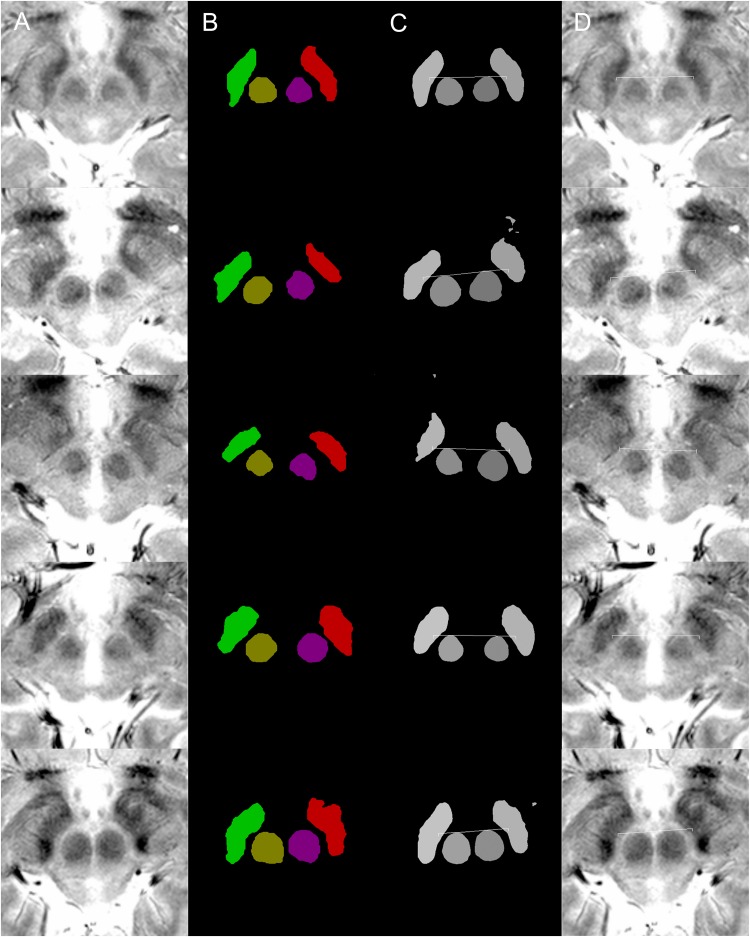
Examples of targeting. Each row of images is from a single patient. **(A)** MRI T2^∗^ fast-acquisition gradient-echo input images. **(B)** Ground truth images of supervised semantic segmentation training or validation. **(C)** Semantic segmentation results and automatic targeting results from a deep learning network trained by 62 non-augmented images. **(D)** Automatic targeting results superimposed on the original image. The automatic targets shown in these figures are for subthalamic nucleus deep brain stimulation. Deep learning-based semantic segmentation and targeting adaptability is shown for various anatomical variations, including right and left asymmetries in the second and third rows, the large inter-red nuclei distance in the fourth row, and large red nuclei in the fifth row.

Axial images from about 4 mm below the anterior–posterior commissure line and showing the mammillothalamic tract were obtained for semantic segmentation training and targeting using Leksell SurgiPlan version 9.0 (Elekta, Stockholm, Sweden) (Figure 1A; Bejjani et al., 2000; Starr et al., 2002; Aviles-Olmos et al., 2014; Park et al., 2017, 2018). MRI images from around the midbrain and basal ganglia were magnified and stored in 500 × 500-pixel RGB jpg format to be processed by the Caffe deep learning tool (Shelhamer et al., 2017).

**FIGURE 2:**
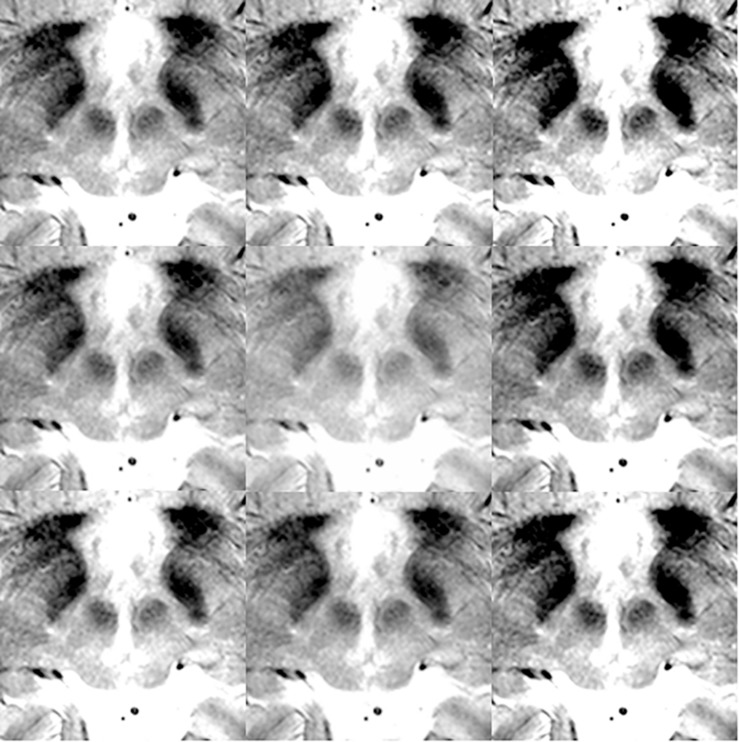
Data augmentation examples for training and validation images.

For the deep learning algorithm used, the necessity of augmentation was known to be low or non-existent when the large and variable VOC-2011-2 dataset was used (Shelhamer et al., 2017). Thus, no positional or rotational augmentation was performed. Instead, we noted that the segmentation results were susceptible to contrasts. Thus, we only performed contrast adjustments and augmentation. For optimal images, the contrast was adjusted in the surgical planning software, Leksell SurgiPlan version 9.0 (Elekta, Stockholm, Sweden), to ensure good visualization of the STN and red nucleus. Next, image brightness preprocessing and data augmentation were performed using Matlab^®^ (Natick, MA, United States). The Matlab image processing toolbox “Imadjust” function was used, with no additional options, to produce images of improved contrast automatically by saturating the upper and lower 1% of pixels. Various image contrast adjustments were then used to augment the original data nine times. Specifically, the low-in and high-in options of the “imadjust” function were set to 0–0.2 and 0.8–1.0, respectively, without low-out or high-out options. Examples of contrast augmentation are shown in Figure 2. Thus, when 102 images were augmented nine times, a total of 918 images were obtained.

**FIGURE 3:**
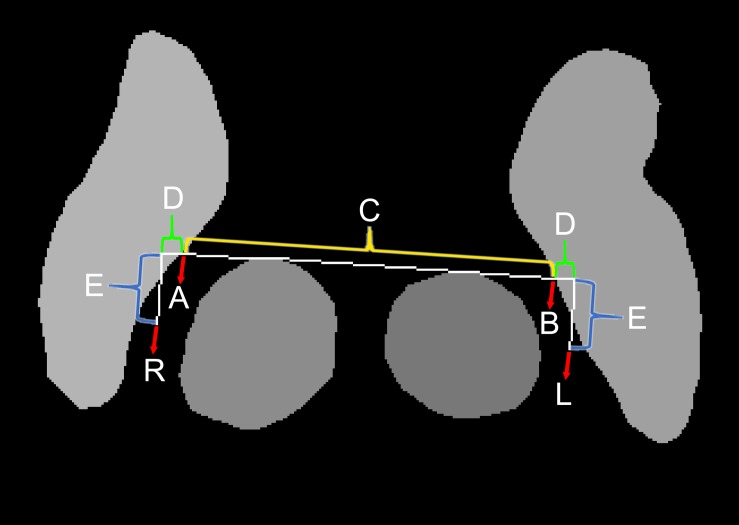
Method for automatic targeting using the semantic segmentation margins.

**FIGURE 4:**
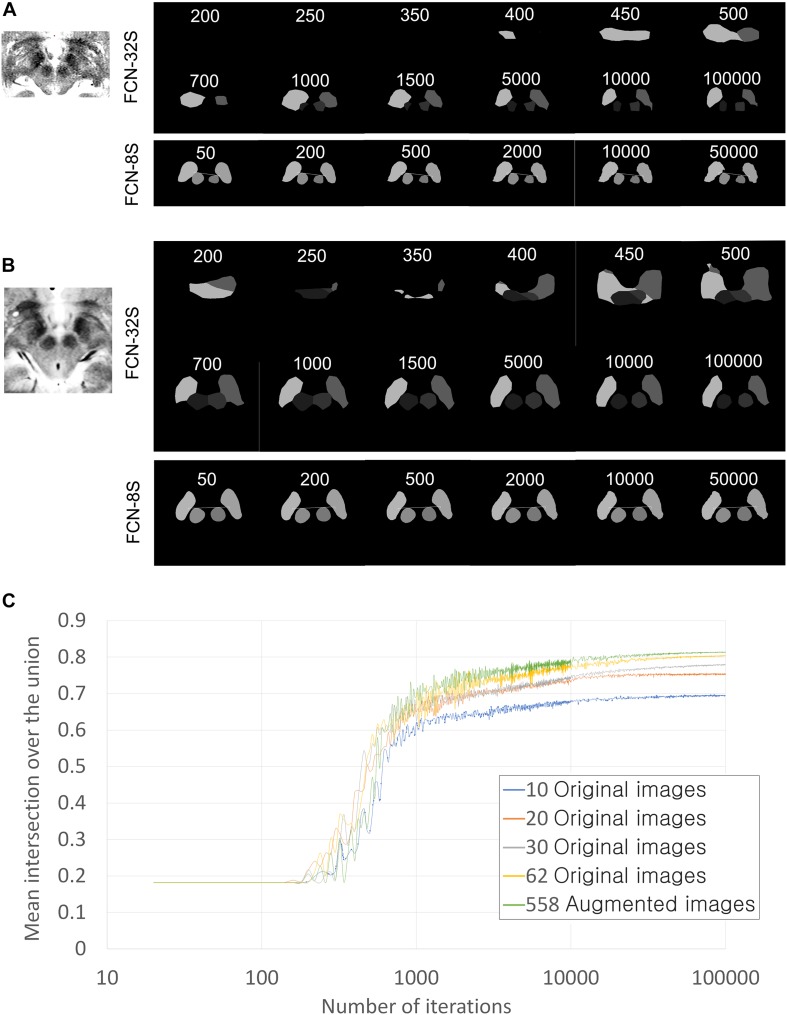
Training progression according to training iterations and training networks. **(A)** Qualitative training progression for the MRI of Patient 1. Upper two rows: training progression images using the FCN-32s coarse segmentation network. Lowest row: training continuation using FCN-8s, which showed the fine segmentation network. Segmentation using FCN-8s has smoother and clearer margins than that using FCN-32s only. **(B)** Training progression for MRI of Patient 2. **(C)** Quantitative improvements in semantic segmentation accuracy in terms of mean intersection over the union (IoU) values according to the number of training iterations and the amount of data used. Validations were performed in 360 augmented images for all graphs. The numbers of iterations are displayed on a log scale for better visualization of improvement curves.

In total, 102 matched T2^∗^ MRI images (Figure 1A), along with ground truth information (Figure 1B), were collected. The ground truth boundaries of the STN and red nucleus were drawn manually. These images were all of equal size (500 × 500 pixels) and were stored in 8-bit indexed color png file formats. Each ground truth image was matched to corresponding MRI images of the same filename and size. Four image classes were used: right STN, right red nucleus, left red nucleus, and left STN. These were filled with different colors to designate the semantic segmentation classes (Figures 1, 3, 4). This ground truth information was used for training, as well as for calculating the accuracy of the validation dataset.

The network used was the VGG-16-derived, semantic segmentation, fully convolutional network (FCN-VGG-16) (Shelhamer et al., 2017). This network was trained using the above-mentioned manually drawn ground truth information for the STN and red nucleus. First, we performed training using FCN-32s, a coarse semantic segmentation network that uses higher layer information, with 100,000 iterations. The FCN-32s-trained Caffe deep learning model was then retrained using 50,000 iterations of the FCN-8s, a fine semantic segmentation network (Shelhamer et al., 2017).

In the section “Results,” two measures of semantic segmentation accuracy are shown: mean accuracy and mean intersection over the union (mean IoU) (Rezatofighi et al., 2019), otherwise known as the Jaccard score (Shelhamer et al., 2017). That is, the mean accuracy is the semantic segmentation accuracy as calculated by averaging the accuracy of all classes (Shelhamer et al., 2017). The mean IoU is the similarity between sample sets, defined as the size of the intersection divided by the size of the union of the sample sets (Rezatofighi et al., 2019).

When ground truth pixels are A and semantic segmentation result pixels are B, IoU was defined as follows:

$$\mathrm{Intersection}\,\mathrm{over}\,\mathrm{the}\,\mathrm{union}\,\left(\text{IoU}\right)=\frac{A\cap B}{A+B-A\cap B}$$

Thus, mean IoU is lower than mean accuracy, and it is considered more important in semantic segmentation studies (Shelhamer et al., 2017; Rezatofighi et al., 2019). For this reason, only mean IoU is shown in the graph (Figure 4C) and is converted to the Dice coefficient (Dice, 1945) for the purposes of comparison with the literature (Choi and Jin, 2016; Visser et al., 2016; Mehta and Sivaswamy, 2017; Milletari et al., 2017; Kim et al., 2019; Figure 4C). The following method was used for the conversion:

$$\mathrm{Dice}\,\mathrm{coefficient}\;=\frac{2\,A\cap B}{\text{A}+\text{B}}=\frac{\frac{2\,A\cap B}{A+B-A\cap B}}{\frac{A+B}{A+B-A\cap B}}=\frac{2\,\frac{A\cap B}{A+B-A\cap B}}{1+\frac{A\cap B}{A+B-A\cap B}}=\frac{2\,I\,o\,U}{1+I\,o\,U}$$

This conversion is exact for single-class IoU, and we performed approximate conversion for the mean IoU of four classes.

**FIGURE 5:**
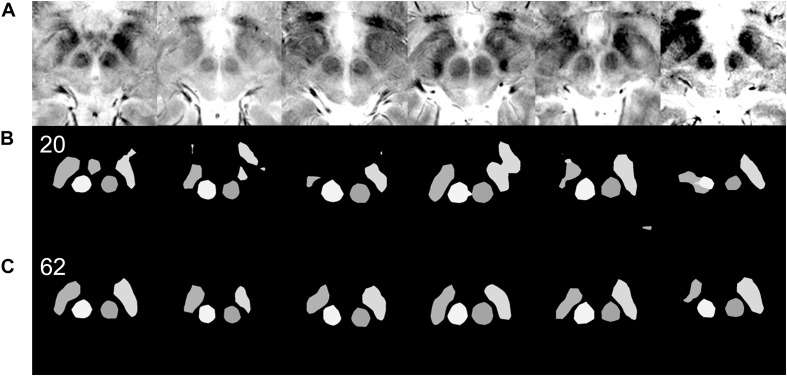
Qualitative differences in semantic segmentation results depending on the amount of training data. Each column of images is from a single patient. Images that are inadequately segmented by the network trained with 20 original images are qualitatively well segmented by the network trained with 62 original images. **(A)** Input MRI T2^∗^ sequence images with various contrast settings. **(B)** Results of semantic segmentation by the coarse segmentation network, FCN-32s, trained with 20 images. Twenty images were not enough for qualitatively and quantitatively accurate semantic segmentation (Figure 4C). **(C)** Results of semantic segmentation by the coarse segmentation network, FCN-32s, trained with 62 images.

**FIGURE 6:**
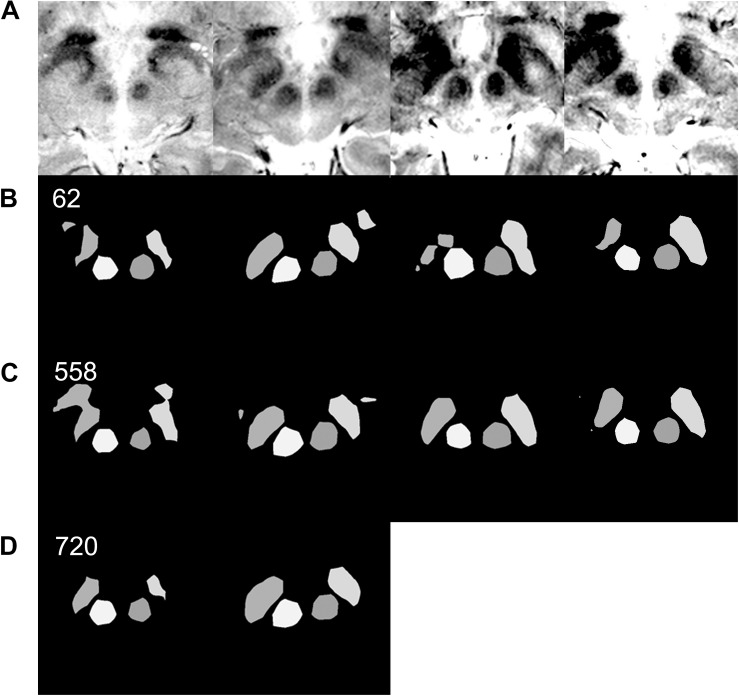
Effect of data augmentation on semantic segmentation quality. **(A)** Input MRI T2^∗^ sequence images with various contrast settings. **(B)** Results of semantic segmentation by the coarse segmentation network, FCN-32s, trained with 62 images. Sixty-two images are sufficient to achieve qualitatively accurate semantic segmentation for most patients (Figure 5). However, examples of inadequate segmentation by the network trained with 62 images are shown in this row. **(C)** Results of semantic segmentation by the coarse segmentation network, FCN-32s, trained with 558 augmented images. In the right two columns, semantic segmentations are qualitatively accurate. **(D)** Results of semantic segmentation by the coarse segmentation network, FCN-32s, trained with 720 augmented images. Semantic segmentation is qualitatively improved in the left two image columns, without segmentation blobs outside of the ground truth.

We also performed qualitative evaluations of the semantic segmentation results manually. When there was a blob or island of wrong segmentation outside of the ground truth or the segmentation territory was substantially smaller, approximately 80% less than the ground truth, we defined the semantic segmentation as being inadequate. Examples of qualitatively inadequate segmentations are shown in row B of Figure 5 and rows B and C of Figure 6.

### Deep Brain Stimulation Imaging Protocols for Clinical Applications

A Leksell Frame G (Elekta, Stockholm, Sweden) was fixed in parallel with the anterior-commissure and posterior commissure considering external landmarks, and imaging followed the DBS protocol (Park et al., 2017, 2018). Bilateral lines crossing the lower margin of the orbit rim and external auditory canal were used as external landmarks during frame applications to verify pitch and lateral tilt angle of frames (Starr et al., 1999). Roll and yaw of frames were maintained parallel with a midline drawn from the nasion to the apex and a horizontal line drawn 3 cm above the eyebrows. Ear bars were also used to facilitate orthogonal frame alignment.

For surgical planning and pre- and post-operative coregistrations and accuracy measurements, Medtronic Stealth Cranial version 3.0.1 was used alongside StealthMerge^TM^ from the Stealth DBS software suite (Medtronic, Dublin, Ireland).

Three-dimensional, magnetization-prepared gradient-echo (3D-MPRAGE) sequences were obtained and used to define the anterior commissure, posterior commissure, and midline.

In both patients, we obtained 3.0-T MRI T2 images 2 mm in thickness using an Achieva 3.0-Tesla MRI Machine and software release 2.1.3.2 (Phillips Healthcare, Amsterdam, Netherlands), with a TR of 3000 ms, a TE of 80 ms, and a flip angle of 90° to determine the margins of the STN and red nucleus before surgery. The field of view was AP (mm) = 300, RL (mm) = 239, and FH (mm) = 100. The voxel size was 0.57 × 0.57 × 2 mm, and the matrix size was 528. In Patient 1, the MRI for targeting was obtained with the stereotactic frame applied, while for Patient 2, the MRI was obtained before stereotactic frame fixation. Preoperative CT images were obtained using a LightSpeed 16-channel CT System (GE Healthcare, Little Chalfont, Buckinghamshire, United Kingdom) with a section thickness of 1.25 mm.

Magnetic resonance imaging without a metal stereotactic frame were coregistered with preoperative, frame-applied stereotactic CT images to calculate the Leksell stereotactic coordinate. Using this process, deep learning-based 3T MRI analysis targeting results could be applied to the Leksell coordinate system for DBS using the stereotactic frame.

After surgery, stereotactic CT images were obtained using the same protocol with the stereotactic frame in both patients. The post-operative stereotactic CT images were coregistered with the preoperative CT images, and the stereotactic error in comparison to preoperative deep learning-based planning was checked in the probe’s eye view (Ellenbogen et al., 2018).

### Deep Learning-Based Targeting

Because deep learning-based semantic segmentation of the STN could be performed at a level that was qualitatively similar to that of humans and at least quantitatively similar to inter-rater variations (Ewert et al., 2019), no further complex machine learning algorithm was required for automatic targeting. Unlike previous automatic targeting algorithms, which have tended to operate based on coregistration or an atlas (Pallavaram et al., 2015), we used the semantic segmented anterior margins of the red nuclei and the borders of the STN, as a human surgeon does (Figure 3; Bejjani et al., 2000).

The automatic targeting method closely mimics the Bejjani method (Bejjani et al., 2000), which is performed by human surgeons after the anatomical structure margins have been determined manually. At first, a horizontal line (C and D) crossing the anterior margins of both red nuclei is drawn (Figure 3). Next, the points where the horizontal line crosses the medial margins of the STN are identified (A and B). In the present study, the distance between A and B was measured and designated as C. Our target was slightly lateral and posterior to points A and B. The targets were calculated as lengths D and E from points A and B. The angle between D and E was 90°. We determined lengths D and E using relative ratios compared with length C. Considering the usual locations of the STN (Daniluk et al., 2010), length C was approximately 20 mm. For the posterior subthalamic area target in Patient 1, the ratio between C and D was 20:2, while the ratio between C and E was 20:4. For the STN target in Patient 2, the ratio between C and D was 20:2 and the ratio between C and E was 20:1. Thus, the posterior STN target (Blomstedt et al., 2009, 2010) is more posteriorly located than the STN target, which is closer to the red nuclei anterior margin line (Foltynie et al., 2011; Aviles-Olmos et al., 2014).

Automatic targeting based on the margins of anatomical structures was performed using a custom-made Python-based program. We defined targeting success in terms of the identification of cross points (A and B in Figure 3) along a line crossing the anterior margins of two red nuclei (Figure 3) and both medial margins of the STN. In contrast, targeting failure was defined as a failure to identify these points. If semantic segmentation is performed incorrectly or if image quality is poor, the red nucleus or STN may contain anomalous shapes (Figure 5B). In this case, targeting failure may occur.

**FIGURE 7:**
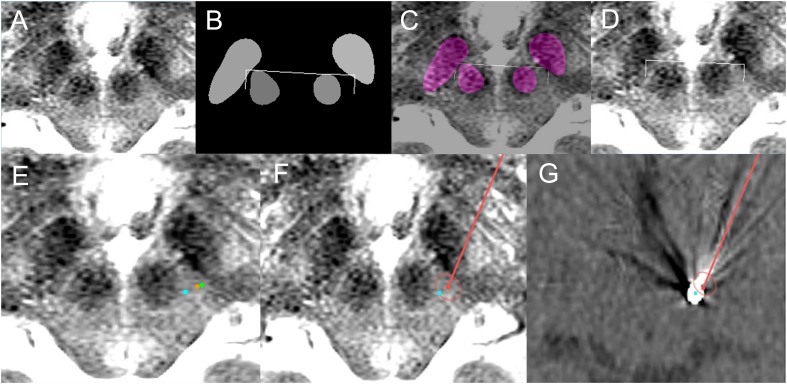
Deep learning-based automatic targeting application for Patient 1. **(A)** Input image for targeting. **(B)** Semantic segmentation results and automatic targeting results. Bilateral ends of the bent edges of the white line are automatic targets calculated using the method shown in Figure 3. **(C)** Semantic segmentation imaging and automatic targets superimposed on the input MRI. **(D)** Automatic targets superimposed on the input MRI. **(E)** Orange dot indicates the deep learning-based automatic target in the left posterior subthalamic area, while the green dot is the indirect target. The cyan dot is the post-operative location of the electrode. **(F)** Planned trajectory and target (red dot and line) and location of the post-operative electrode (cyan dot) superimposed on the preoperative MRI. **(G)** Planned trajectory and target (red dot and line) and location of the post-operative electrode (cyan dot) superimposed on the post-operative CT image.

**FIGURE 8:**
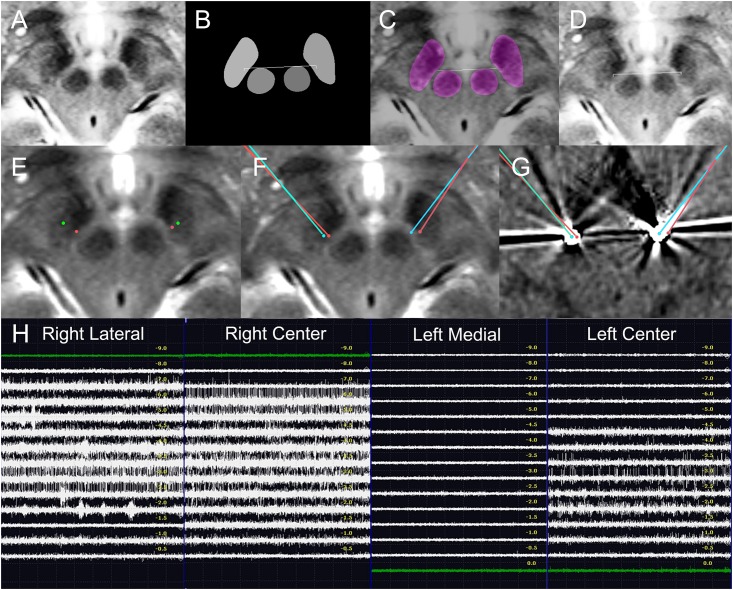
Deep learning-based automatic targeting and application in Patient 2. **(A–D)** Figure parts are arranged similarly to Figure 4. **(E)** Bilateral indirect targets (green dots) and bilateral deep learning-based targets (red dots). Talairach coordinates of bilateral indirect targets are shown for comparison. **(F)** Planned bilateral trajectories and targets (red points and lines) and the bilateral post-operative electrode (cyan dot) superimposed on the preoperative magnetic resonance image. **(G)** Planned bilateral trajectories and targets (red dots and lines) and the bilateral locations of post-operative electrodes (cyan dots) superimposed on post-operative CT images. **(H)** Intraoperative microelectrode recordings.

Indirect targets, i.e., targets determined within Talairach coordinates from anatomical markers, the anterior commissure, posterior commissure, and midline, were not used preoperatively for targeting, and only deep learning-based targeting was used (Kochunov et al., 2002). However, indirect targets have been shown for comparison (Figures 7E, 8E). For Patient 1, the indirect target was lateral: −12.5 mm, anterior–posterior: −6.5 mm, and vertical: −4 mm from the midcommissural point based on Talairach coordinates (Figure 7E). The indirect target for Patient 2 was lateral: ±12 mm, anterior–posterior: −2.5 mm, and vertical: −4.0 mm.

For clinical applications, Medtronic Stealth Cranial version 3.0.1 included in the Stealth DBS software suite (Medtronic, Dublin, Ireland) was used to capture images for targeting. Deep learning-based targeting was performed using these images. After the targets had been determined, they were overlaid onto the same pixels of the input image under maximum magnification using the surgical planning software. The Leksell coordinates of the targets were identified and calibrated, taking into account systematic stereotactic errors (Holl et al., 2010; Park et al., 2018), and numerically rounded up to the nearest 0 or 0.5.

### Operation Procedures, Intraoperative Microelectrode Recordings, and Macrostimulations

Both patients were operated on under local anesthesia. C-arms were used intraoperatively to check electrode positions. For Patient 1, microelectrode recording was not performed because the posterior subthalamic area was not a target with a conspicuous microelectrode recording signal (Plaha et al., 2008; Blomstedt et al., 2009, 2010). Thus, for Patient 1, a microdrive and Ben’s gun, a microdrive component with five electrode passage holes to select the electrode track (Pollak et al., 2002), were not used. Instead, the macrostimulation effect was checked using a portable stimulator.

For Patient 2, bilateral microelectrode recordings were performed using a microTargeting^TM^ microdrive (FHC Inc., Bowdoin, ME, United States) and Ben’s gun (Hariz, 2002; Amirnovin et al., 2006; Shamir et al., 2019). Two tracks of microelecrodes were used for each side. Intraoperative macrostimulation using microtargeting^TM^ microelectrodes was also performed. Stimulations were performed using 1–4 volts.

Using electrical stimulation, the effects of stimulation, including tremor, rigidity, or bradykinesia reduction were assessed by physical examination. Side effects, including eyeball deviations, dystonia, paresthesia, and speech disturbances, were checked.

Medtronic 3389 DBS electrodes (Medtronic, Dublin, Ireland) were used. For both patients, Activa SCs (Medtronic, Dublin, Ireland) were implanted posterior to the pectoralis major muscles. After implantation, the functions and resistances of the DBS systems were checked before wound closure.

## Results

### Semantic Segmentation and Targeting Results

Semantic segmentation was able to adapt to anatomical asymmetry and individual variation (Figure 1). In addition, the algorithm has good regularization characteristics, and the margins of the anatomical structure were smoother (Figure 1C) than in the more irregular training ground truth dataset (Figure 1B). Non-augmented data from 62 patient images were used to produce Figure 1. Later, after increment and augmentation of this data, the semantic segmentation results further improved, and the minor speckles shown in Figure 1C disappeared.

As the iteration progressed, qualitatively ambiguous and intermixed segmentations were progressively more accurately classified, and semantic segmentation accuracy increased (Figures 4–6). Semantic segmentation using FCN-8s produced qualitatively smoother segmentation borders than FCN-32s (Figures 4A,B), as expected based on the network characteristics (Shelhamer et al., 2017). As we increased the number of training images, the semantic segmentation accuracy metrics and quality increased progressively (Figures 4C, 5, 6).

The mean accuracy of semantic segmentation after FCN-32s training increased, as shown in Table 1. The mean IoU is also shown in Figure 4C. In total, 918 augmented training and validation images were used.

**TABLE 1:** Semantic segmentation accuracy improvements according to the number of training images in the validation dataset with 360 augmented images.

**Number of training images**	**Mean accuracy**	**Mean intersection over the union**	**Approximate Dice coefficient converted from mean intersection over the union^∗^**
10 original images	0.770	0.673	0.804
20 original images	0.837	0.757	0.861
30 original images	0.876	0.778	0.875
62 original images	0.904	0.804	0.891
558 augmented images from 62 original images	0.911	0.813	0.897
720 augmented images from 80 original images^†^	0.912	0.821	0.902

*^∗^Dice coefficient = $\frac{2\,I\,o\,U}{1+I\,o\,U}$ when supposing that all class accuracies were identical. IoU mean intersection over the union. ^†^Among a total of 918 augmented training and validation images, the remaining 198 augmented images were used for validation.*

When we converted the best 0.821 mean IoU into a Dice coefficient (Dice, 1945), the speculated approximate mean Dice coefficient was 0.902 (Table 1), supposing that all class accuracies were identical.

Qualitatively, when 40, 50, or 62 training images were used, 17, 28, or 3 of the 360 augmented validation images (4.7, 7.8, or 0.8%) were inadequately segmented, respectively. When 558 or 720 augmented images were used, none of the images were inadequately segmented.

Targeting failure only occurred in 1 of 17 cases when data from 50 patients were used. When training images from 62 or more patients were used, targets could be identified in all 40 of the patient validation images that were available before data augmentation (Figure 5). Thus, when the network was trained using the data from 62 patients, we began clinical application after manual confirmation of the deep learning-based target. The total number of patients from which the training and validation data were obtained was 102; these images were then augmented nine times to yield a dataset of 918 images (Figures 4C, 6).

### Prospective Clinical Applications

The deep learning-based targeting and clinical applications were pre-planned before surgical application. They were then prospectively applied to surgery after manual confirmation of target applicability. Before DBS surgeries, we manually confirmed that the safety of deep learning-planned targets was acceptable for clinical applications following institutional review board-approved protocols.

### Patient 1

The deep learning-based automatic targeting method for the posterior subthalamic area was first applied to a 74-year-old woman with essential tremor on May 24, 2018 (Figure 7).

The patient’s tremor grade was III in both hands before surgery, and her score on the essential tremor rating assessment scale was 35. The deep learning Caffe model network trained using 62 patient images was used, and targeting was performed immediately before surgery after frame-fixed MRI images had been acquired.

Table 2 shows the Talairach coordinates of the deep learning-based targeting, as well as the Leksell coordinates after they were rounded up to the nearest 0.5 or whole number.

**TABLE 2:** Stereotactic coordinates of deep learning targets and post-operative electrode locations.

		**Directions**	**Patient 1 left**	**Patient 2 right**	**Patient 2 left**
			**posterior**	**subthalamic**	**subthalamic**
			**subthalamic area**	**nucleus**	**nucleus**
Talairach coordinate from midcommissural point	Deep learning-based target	Lateral	−11.3	9.2	−10.79
		A–P	−6.7	−4.3	−3.38
		Vertical	−3.7	−4.5	−4.12
	Post-operative electrode	Lateral	−9.5	10.3	−8.86
		A–P	−7.6	−4.2	−3.52
		Vertical	−3.7	−3.1	−4.15
Leksell coordinate	Deep learning-based target	X	113.5	92.0	112.0
		Y	84.5	91.0	92.5
		Z	110.5	110.0	109.5
	Trajectory	Ring	67	77.0	71
		Arc	107.5	67.0	108
	Post-operative electrode	X	111.6	90.9	110.1
		Y	83.2	90.7	92.3
		Z	110.7	108.6	109.5
Stereotactic errors			1.6	0.8	1.7
					

The lead implantation was performed under local anesthesia by S-CP. In particular, left unilateral posterior subthalamic DBS was performed to the deep learning-planned target position. During surgery, the patient’s right hand tremor was checked; it decreased from grade II to grade I after bipolar electrical stimulation using the zero electrode as the anode and the 3rd electrode as the cathode. No hemorrhage or infection occurred.

After surgery, the patient’s tremor had decreased from grade III to grade I, and her essential tremor rating assessment scale score had decreased to 20. Electrical stimulation on the 2nd electrode caused the tremor to be reduced further, and patient follow-up over 8 months was uneventful. The final optimal stimulation setting was as follows: bipolar electrode, 2–1+ with 1.5 V, 60 μs, and 130 Hz stimulation frequency.

### Patient 2

This patient was a 71-year-old woman with Parkinson’s disease who underwent surgery on December 4, 2018. She had been prescribed levodopa and other medications since 2004 to treat her motor disorder, and she underwent MRI in 2013 that showed no abnormality or severe brain atrophy. She underwent FP-CIT-PET in January 2018, which showed bilateral decreased dopamine transporter binding in the putamina and caudate nuclei, with rostrocaudal and ventrodorsal gradients. The probable clinical and FP-CIT-PET diagnosis was Parkinson’s disease.

Before surgery, the patient’s most severe Unified Parkinson’s Disease Rating Scale (UPDRS) motor score in the “medication off” state was 37, while her score in the “medication on” state was 21. Her bilateral rigidity and tremor were minimal, but bradykinesia and gait disturbances were major symptoms.

Because our targets were selected using deep learning algorithm-based automatic targeting, manual indirect or direct targeting methods were not used. Instead, indirect targets are shown for comparison (Figure 8E).

The deep learning network model trained by 558 augmented images and validated using 360 augmented images was used for targeting. Table 2 shows the deep learning-planned target coordinates and actual post-operative electrode locations.

The MRI image for targeting was obtained before stereotactic frame fixation using a 3T T2 MRI sequence 2 mm in thickness. Deep learning-based targeting was performed 1 day before the surgery.

The lead implantation was performed under local anesthesia by S-CP. The left side was operated on first. On the left side, center and medial tracks were used. Our target was close to the medial margin of the STN, and the STN signal was only recorded in the center electrode. The length of the microelectrode passage that showed the STN signals is depicted in Figure 8H. The microelectrode recording showed that the STN extended from −4.5 to −1.0 mm in the left center track. The electrode was positioned in the center track, with the target at a depth of +0 mm.

On the right side, the center and lateral tracks were used, and the STN signal was recorded from −6.0 to −1.0 mm on the right center track and on the lateral track. The electrode was positioned in the center track −1 mm from the target depth. Because deep learning-based targets are located close to the center target, center-track microelectrode recordings with STN signals directly support that the deep learning-based targets were correctly located within the STN. During surgery, electrical stimulation improved the patient’s bradykinesia.

The stereotactic errors are shown in Table 2 and Figure 8. No hemorrhagic or infectious complications occurred.

The patient’s post-operative UPDRS motor score, without stimulation and under minimal levodopa medication (300 mg a day), was 15. Later, her levodopa dose was increased to 450 mg a day, and pramipexole was added. Her UPDRS motor score was 13 without stimulation under medication. Electrical stimulation was turned on after 1 month. After surgery, the most effective electrodes were right 3 (2.5 V, 80 Hz, 60 μs pulse width) and left 3 (1.5 V, 80 Hz, 60 μs). At the best stimulation setting and with the correct medication, the patient’s UPDRS motor score was 7. Under right stimulation, her left bradykinesia improved from grade 2 to grade 1. Under left stimulation, her right bradykinesia improved from grade 2 to grade 1. Her gait disturbances improved from grade 1 to grade 0, and she showed no complications over 3 months of outpatient follow-up visits.

## Discussion

### Deep Learning-Based Semantic Segmentation and Targeting Characteristics

Recent state-of-the-art adaptive atlas-based studies using 3T or 7T MRI reported a Dice coefficient of 0.64–0.67 in the STN (Ewert et al., 2019; Kim et al., 2019), and another study indicated that the red nucleus is easier to segment, with an approximate 0.85 Dice coefficient (Visser et al., 2016). Thus, we speculate that the mean Dice coefficient, including both the STN and the red nucleus, may have been about 0.75–0.77 in previous studies (Visser et al., 2016; Ewert et al., 2019; Kim et al., 2019). A recent two-dimensional deep learning study had a mean Dice coefficient of 0.85 for basal ganglia structures on MRI (Mehta and Sivaswamy, 2017). However, this study did not segment the STN and red nucleus (Mehta and Sivaswamy, 2017). The Dice coefficient-converted accuracy in the present study (∼90.2%) (Table 1) was numerically higher than those of previous non-deep learning studies (Visser et al., 2016; Mehta and Sivaswamy, 2017; Milletari et al., 2017; Ewert et al., 2019; Kim et al., 2019). The present study involved a two-dimensional image analysis similar to a recent fast and memory-efficient deep learning study (Mehta and Sivaswamy, 2017). However, a few non-deep learning studies were three-dimensional (Ewert et al., 2019; Kim et al., 2019). Thus, these results should be compared with caution.

Various analytical factors differed between previous studies and the present study (Visser et al., 2016; Mehta and Sivaswamy, 2017; Milletari et al., 2017; Ewert et al., 2019; Kim et al., 2019). Thus, we cannot ascertain which algorithms are superior. Considering the moderately different results from the other deep learning studies, we speculate that not only the algorithm but also MRI image quality control, sequence types, focused magnification, and high image contrasts influenced accuracy.

Because our deep learning-based surgical targeting achieved high accuracy, we could apply it in real clinical patients and conduct manual verification. Individual anatomical variability has been a major obstacle to atlas- and coregistration-based automatic targeting methods (Ashkan et al., 2007; Patel et al., 2008; Daniluk et al., 2010; Xiao et al., 2014; Pallavaram et al., 2015). The center positions of the STN are highly variable, fluctuating over 3–6 mm depending on the axis direction (Daniluk et al., 2010). In addition, the shape, size, and position of the STN may be asymmetric, variable, or irregular, as shown in Figure 1. Thus, atlas-based or coregistration-based methods may inevitably be inaccurate due to individual differences in the atlas or other structures (Ewert et al., 2019; Kim et al., 2019; Shamir et al., 2019). Non-deep learning adaptive algorithms, as well as semantic segmentation studies of the basal ganglia and brainstem structure, have used simpler image features than deep learning (Visser et al., 2016; Shamir et al., 2019). It follows that deep learning may be more adaptive to anatomical variation and better at generalization using more abstractive features (Figure 1). Thus, the final segmentation accuracy may be higher when deep learning is used.

### Training and Validation MRI Image Protocol Selection

Image quality was crucially important to ensure the best performance for the algorithm in the present study. Various MRI sequences, including T2- (Bot et al., 2016), 3T T2^∗^- (O’Gorman et al., 2011; Kerl et al., 2012a, b), and T2^∗^- (Lefranc et al., 2014; Nagahama et al., 2015) weighted angiography, susceptibility-weighted imaging (O’Gorman et al., 2011; Polanski et al., 2015), and fluid-attenuated inversion recovery imaging (Heo et al., 2015) have been used for STN segmentation or posterior subthalamic area targeting. All of these are probably practical for clinical use. Because the STN has a high iron content, susceptibility-weighted sequences are beneficial for STN visualization (Polanski et al., 2015). However, excessive susceptibility artifacts can distort and exaggerate the STN size and margins, so the accuracy of susceptibility-weighted image margins has been questioned (Bot et al., 2016). Thus, to balance visibility, measurement accuracy, familiarity for many users, and broad applicability, we selected the T2^∗^ sequence, which is similar to the frequently used T2 sequence and has moderate susceptibility weighting capabilities. In addition, the 3T MRI used in the present study with or without frames may be better for visualization and direct targeting of DBS than is stereotactic 1.5T MRI (Cheng et al., 2014; Southwell et al., 2016).

Distortion of 3T MRI with a frame is still being investigated, and it is not yet fully confirmed for stereotactic use (Neumann et al., 2015). Thus, coregistration between 3T MRI and CT was required (Neumann et al., 2015).

Recently, the cross-center reproducibility of deep learning algorithms has been an important issue (Zech et al., 2018). Algorithms require matched or similar preoperative MRI sequences of the best possible quality to ensure accurate performance. In the present study, the T2^∗^ MRI sequence used in training and validation was unavailable in the hospital in which the clinical application was carried out. Thus, a T2 MRI sequence was used for targeting in the hospital. T2 and T2^∗^ mostly share similar image contrasts, and we could perform cross-MRI sequence application of the algorithm (Figures 7, 8). We partly showed that cross-center and cross-MRI-sequence application of T2-related MRI sequences with similar image contrast values and acceptable quality was possible.

However, MRI sequences with worse quality cannot be used. Worse MRI image contrast due to lower magnetic field (Cheng et al., 2014; Southwell et al., 2016), 1-mm thickness T2 slicing, and higher noise from metal artifacts or any cause (O’Gorman et al., 2011) can result in a lower contrast-to-noise ratio, which is not suitable for machine learning algorithm application.

In this regard, the inclusion of lower quality images during training may improve algorithm robustness toward lower quality images. However, image quality can be controlled preoperatively to ensure the best outcomes for DBS. Thus, we suggest that algorithms will perform better if poor quality images are not used.

### Deep Learning Network and Training Characteristics

The semantic segmentation accuracy of fully convolutional neural networks has been reported based on the VOC-2011-2 dataset, which has 20 classes and 11,530 images containing 27,450 annotated objects (Shelhamer et al., 2017). The present study had fewer classes, and the shapes of each class may have been less variable than in the VOC-2011-2 dataset. However, the mean IoU in the present study was approximately 15% higher than in VOC-2011-2 (82.1% vs. about 67%), even though the same network architecture was used (FCN-VGG-16) (Shelhamer et al., 2017). We speculate that our study showed high accuracy because we used fewer classes with more constant shapes and positions and with less structural variability and used grayscale images. High contrast anatomical structures in T2^∗^ MRI sequences are also probably related to high validation accuracy.

Similar to a previous report, only a short amount of time was required in the present study for a single iteration and semantic segmentation – about 250 ms (four images processed per second). This time is related to the size of the deep network and the number of VGG16-based network parameters. The speed in the present study was fast enough to allow clinical application without delay during the procedure.

When we expanded the data to include over 50 images, the accuracy only improved slightly and then plateaued (Figure 4C). Thus, increasing the number of images is not likely to further improve the segmentation accuracy. Considering that the mean IoU was over 80% in the present study – a very high semantic segmentation accuracy (Figure 4C) – we concluded that qualitatively and quantitatively sufficient training data were used to allow four-class semantic segmentation. Thus, deep learning-based semantic segmentation of basal ganglia is possible with a smaller number of samples than the VOC-2011-2 dataset – 62 images for four-class classification.

In a previous semantic segmentation study that used the VOC-2011-2 dataset, data augmentation yielded no improvements in semantic segmentation results (Shelhamer et al., 2017). However, in the present study, a small increase in accuracy was noted (Figure 4C). It follows that data augmentation using variable contrast data improves semantic segmentation adaptiveness when using various contrast settings. The differences may be that the VOC-2011-2 dataset was already highly variable and that data comprising a higher number of images no longer required augmentation.

### Deep Learning-Based Targeting, Direct and Indirect Targets, and Targeting Standardization

There are two kinds of deep learning-based targeting methods: direct and indirect. Direct targets are based on MRI anatomical structure margins (Holl et al., 2010; Foltynie et al., 2011; Aviles-Olmos et al., 2014). Because our deep learning-based targeting was also performed based on MRI anatomical structure margins, our deep learning-based targeting method can be considered a type of MRI-based direct targeting method (Bejjani et al., 2000). Indirect targeting is based on Talairach coordinates calculated from the anterior commissure, posterior commissure, and midline (Kochunov et al., 2002; Tu et al., 2018). The present study did not identify these anatomical points, and as such, our deep learning-based method is not a substitution for indirect targets. Nonetheless, indirect targets were manually determined and are shown for comparison purposes (Figures 7, 8).

The exact locations of DBS targets can vary among surgeons, and DBS centers and best locations are controversial. Some surgeons target the center of the STN (Bejjani et al., 2000), while others target more medial sites (Toda et al., 2009), medial–posterior sites, or other locations (Aviles-Olmos et al., 2014). We used targets that were close to the medial margin of the STN and slightly posterior to the anterior margins of the red nucleus (Foltynie et al., 2011; Aviles-Olmos et al., 2014). This target is similar to that used in a previous image-guided DBS study (Aviles-Olmos et al., 2014).

Indeed, individual surgeons may not be consistent in this regard. Thus, the location of a quantified point or line (e.g., 1 mm lateral from the medial border of the STN) may vary among individual surgeons when targeting is manually applied. However, when identical deep learning algorithm models and input images are used, targeting is completely identical and can be standardized without subjective differences.

The results of different DBS-related clinical trials may be influenced by target variations among surgeons. Deep learning-based automatic segmentation and targeting would be more consistent and regular than manual methods. Thus, deep learning DBS may allow standardization of targeting locations in multi-center clinical trials involving multiple surgeons. Therefore, deep learning-based targets may be used to aid manual targeting, similar to indirect targets, for better standardization of targets.

In the near future, the exact targeting location may also be automatically optimized based on post-operative outcomes using the deep learning-based targeting observed in the present study as well as the outcome-guided machine learning recently reported from our group (Park and Chung, 2018).

Once trained, deep learning-based targeting does not need to be retrained or re-experience an early learning curve during clinical applications in the same way that trained human surgeons do. Deep learning-based targeting can be applied in the fully trained state from large-scale anatomy-electrode location-outcome data. Thus, consistently high targeting quality can probably be achieved with potentially low cost.

### Related Studies

Deep learning-based morphometry of basal ganglia structures is related to the present study (Mehta and Sivaswamy, 2017; Milletari et al., 2017). A recent study reported microelectrode recording verification of a machine learning, active shape modeling-based segmentation of the STN (Shamir et al., 2019). Another recent report did not involve segmentation-classified deep learning networks to select image patches and reported good DBS post-operative outcomes (Bermudez et al., 2018). A recent conference proceeding reported microelectrode recording signal analysis using a deep neural network (Guillén-Rondon and Robinson, 2016). Recently, biopsy needle or catheter position was analyzed with deep learning in relation to image-guided surgery or interventions (Mehrtash et al., 2019; Paolo et al., 2019). However, none of these studies are directly related or applicable to deep learning-based automatic targeting.

### Limitations

Because the present study is the first application of deep learning-based targeting for DBS, the algorithm and clinical applications were made as simple as possible. Thus, there are many avenues for further investigations and development. First, the algorithm uses a two-dimensional axial slice image only. Selecting the main Bejjani target point by deep learning is possible only by using a single axial image (Bejjani et al., 2000). The subsequent trajectory planning processes considering upper and lower T2 MRI axial images or three-dimensional anatomies were performed manually. The selection of the axial image for targeting was manual, and this step determined the targeting depth.

Second, we only used 3T MRI data, and we found that the applicability of 1.5T or poor-quality MRI images was qualitatively inadequate (not shown). The algorithm may be further improved by including lower quality MRI training data for increased robustness in various clinical settings. However, DBS preoperative MRI quality can be electively controlled to be optimal in most centers, and the use of only high-quality MRI is also a good option. In the present study, relative distances from semantically segmented anatomical structures and the deep learning-based targets were manually adjusted to mimic targets used by neurosurgeons. However, the exact target location can be improved using information about targets via an outcome-guided machine learning method (Park and Chung, 2018). The number of clinical applications in the present study is few. Further investigations into more clinical applications of this technique are warranted.

## Conclusion

Using a deep learning algorithm and 3T MRI data magnified for midbrain structures, we achieved high semantic segmentation accuracy that was adaptive for anatomical variability of the STN and red nucleus. We could automatically determine the DBS target from the segmented anatomical structures. The deep learning-based target could be applied in real patients successfully without target modification and electrode track change. This study is the first to show that deep learning-based DBS surgical planning is clinically applicable in image-guided surgeries. A deep learning-based targeting technique is potentially more objective, consistent, and analyzable than manual methods. Deep learning-based DBS targeting can potentially be improved for better outcomes in the near future.

## Data Availability Statement

The datasets generated for this study are available on request to the corresponding author.

## Ethics Statement

This study was pre-approved by the institutional review boards of Gangneung Asan Hospital (2018-07-22) and Asan Medical Center (S2016-1230-0005). Before the clinical application, all patients and families signed informed consent forms that had been approved by the institutional review boards.

## Author Contributions

S-CP designed the study and mediated correspondence regarding the manuscript, applied for and acquired a national research foundation grant, wrote the manuscript and made all the figures, participated in training DBS dataset patient surgeries, guided training through ground truth dataset drawing, designed and verified the training and validation dataset, modified the open-source Caffe deep learning semantic segmentation program and performed validation dataset analysis with various amount of data up to 918 images, performed data augmentation and tested performance, designed the targeting method, recruited Patient 1, performed clinical application of DBS, and verified the DBS target before clinical application. JC adapted the open-source Caffe deep learning semantic segmentation program for the present study, performed the initial training with data on 62 patients, and designed the Python-based targeting program. SL constructed the ground truth training dataset and collected and arranged the data. WJ performed the clinical application, patient evaluation, and management, and recruited Patient 2. CL and JL established the training dataset. CL performed the patient recruitment and perioperative management for the training dataset patients. JL performed the operations, and set up MRI and DBS protocols for the training dataset patients.

## Conflict of Interest

The authors declare that the research was conducted in the absence of any commercial or financial relationships that could be construed as a potential conflict of interest.
